# Effect of silver nanoparticles and *Bacillus cereus* LPR2 on the growth of *Zea mays*

**DOI:** 10.1038/s41598-020-77460-w

**Published:** 2020-11-23

**Authors:** Pankaj Kumar, Vikas Pahal, Arti Gupta, Ruchi Vadhan, Harish Chandra, Ramesh Chandra Dubey

**Affiliations:** 1 Department of Microbiology, Dolphin (PG) College of Science and Agriculture, Fatehgarh Sahib, Chandigarh, Punjab 140307 India; 2 Department of Microbiology, Dolphin (PG) Institute of Biomedical and Natural Sciences, Dehradun, Uttarakhand 248007 India; 3 Department of Zoology, Sri Avadh Raj Singh Smarak Degree College, Gonda, Uttar Pradesh India; 4 Department of Botany and Microbiology, Gurukula Kangri Vishwavidyalaya, Haridwar, Uttarakhand 249404 India

**Keywords:** Microbiology, Plant sciences, Nanoscience and technology

## Abstract

The effect of Plant Growth Promoting Rhizobacteria (*Bacillus* sp.) and silver nanoparticles on *Zea mays* was evaluated. The silver nanoparticles were synthesized from *Tagetes erecta* (Marigold) leaf and flower extracts, whereas PGPR isolated from spinach rhizosphere. The silver nanoparticles (AgNPs) were purified using ultra centrifugation and were characterized using UV–Vis spectroscopy at gradient wavelength and also by High Resolution Transmission Electron microscopy (HRTEM). The average particles size of AgNPs was recorded approximately 60 nm. Almost all potential isolates were able to produce Indole Acetic Acid (IAA), ammonia and Hydrogen cyanide (HCN), solubilized tricalcium phosphate and inhibited the growth of *Macrophomina phaseolina *in vitro but the isolate LPR2 was found the best among all. On the basis of 16S rRNA gene sequence, the isolate LPR2 was characterized as *Bacillus cereus* LPR2. The maize seeds bacterized with LPR2 and AgNPs individually showed a significant increase in germination (87.5%) followed by LPR2 + AgNPs (75%). But the maximum growth of root and shoot of maize plant was observed in seeds coated with LPR2 followed by AgNPs and a combination of both. *Bacillus cereus* LPR2 and silver nanoparticles enhanced the plant growth and LPR2 strongly inhibited the growth of deleterious fungal pathogen. Therefore, LPR2 and AgNPs could be utilized as bioinoculant and growth stimulator, respectively for maize.

## Introduction

The global human population is increasing day by day. As per the report published by the Food and Agriculture Organization^1^, there will the immense demand of agriculture and agricultural-based products in the future, resulting in dietary changes. To fulfill the needs of food for all the substantial additional agricultural production of 2.4 × 10^9^ t/year is required. Agriculture is a very important component of the environment and is influenced by an interaction between humans and nature resulting in an alteration in climate and environment. The continuous use of a chemical fertilizers has resulted in the ultimate changes in the pools, soil nutrients, which are important factors for growth promotion because crop yield and quality of food is highly dependent on fertilizers and quality of the agricultural lands^2^.

Maize or corn (*Zea mays*) belongs to the family *Poaceae* and genus *Zea* is the most important cereal in the world after wheat and rice. Globally, maize is known as queen of cereals because it has the highest genetic yield potential among the cereals. It possesses a high nutritive value and is important as a coarse grain. Maize is used as a staple food of human, a livestock feed, a raw material for more than 3500 products such as QPM (Quality Protein Maize), infant foods, starch, alcohol, textile and medicinal products, etc^3–6^. The present world production of maize is about 1099.61 million metric tons, which is highest as compared to wheat and rice^7^. It is estimated that corn will be the developing world’s largest crop by 2025, and between now and 2050 the demand for maize in the developing world is expected to double^8^. In India, maize is one of the important cereals cultivated throughout the year (current annual production is about 28 million metric tons) for various purposes including grain, fodder, green cobs, sweet corn, baby corn, pop corn in peri-urban areas^9^. Similar to other crops, maize production is also negatively affected by various types of insects and fungal diseases that pose threats to maize yield. Fungal infection caused mainly by *Fusarium graminearum*, *Stenocarpella maydis*, and *Macrophomina phaseolina* are among the principal causes of deterioration and loss of corn grain^10^. The worldwide yield losses due to various diseases in maize crops have been estimated approximately 12–40%^11^.

In sustainable agriculture, the crops should be disease resistant, tolerant to salt, droughts, heavy metal stresses and especially have good nutritional value. To fulfill these properties, we have a good alternative to chemical fertilizers, which are soil resident microorganisms (bacteria, fungi, algae, etc.) that enhance the nutrient uptake capacity. Among these potential soil microorganisms, bacteria are more beneficial than the others because they can tolerate almost all types of biotic and abiotic stresses, present in huge amounts in agricultural soil. Such type of beneficial bacteria present in the rhizospheric region of plant are known as Plant Growth Promoting Rhizobacteria (PGPR)^12^. In this way, PGPR can be a better alternative to enhance the plant health, increase yield, and control phytopathogens without causing any environmental contamination^13,14^. PGPR enhance the growth of crops either by the growth substances produced by them or by up taking the nutrients from the environment and supply to the plant. Actually, PGPR help plant growth by a combination of physiological attributes such as asymbiotic N_2_ fixation^15^, phytohormones production namely indole-3-acetic acid (IAA), cytokinin, gibberellins^16^, solubilizing insoluble mineral phosphate, zinc and potassium, etc.^17^, siderophore production^18^ and by inhibiting the deleterious fungal phytopathogens^14,19,20^.

Several PGPR have been isolated worldwide and some of them have been commercialized, containing the species of *Azobacter*, *Azosprillum, Bacillus*, *Enterobacter*, *Klebsiella*, *Pseudomonas*, *Serratia* and *Variovorax*^21^. Among them, *Bacillus* spp. possess good tolerance properties in adverse conditions, hence they are better for several crops^22^. In the current scenario, the natal approaches are generally used alternately to chemical fertilizers for the better improvement in crop yield, and also to include plant nutrient management systems. A potential PGPR must colonize the rhizosphere, enhance the growth of plants, have multi spectrum mode of action, ecofriendly, good survivality and tolerant to heat, UV radiation, various oxidizing agents and other adverse ecological conditions.

Besides PGPR, nanotechnology is also a good option to develop eco-friendly compounds in place of chemical fertilizers/ pesticides^23,24^. Nanotechnology has refined the current economy in different ways as efficiency, safety, patient loyalty, eventually sinking health care costs, etc. and the detection, treatment of infections, nutrient deficiency, and other health problems are also accomplished by nanoscale devices^25^. The products derived from nanotechnology are colloidal in nature and known as nanomaterials because of smaller particle size.

The size of nanoparticles (NPs) varies from 5 to 100 nm and owing to the high surface-to-volume ratio they were found to possess unique and novel physico-chemical properties that were observed to be superior in comparison to their bulk counterpart^25^. Besides their importance in various fields of science and technology, NPs have now revolutionized the agriculture fields with respect to fabrication, storing, packaging, processing, and transportation of agricultural produce^26^. Recently, nanotechnology derived fertilizers magnetize the attention of agriculture scientist throughout the world. Nano-molecule of fertilizer can be encapsulated or entangled inside the hollow nanomaterials or nanocomposites with an outer protected polymer film or delivered directly in the field as NPs or in the form of emulsions. As the nanotechnology based fertilizers have a favorable effect on agriculture produce, this resulted in a favorable impact on both economy and the environment^27^.

The use of nano-sized silver particles (AgNPs) as antimicrobial agents has become more common as technology advances, making their production more economical. AgNPs have been well documented for their profound antimicrobial effect against diverse categories of microbes and hence are being used in controlling of various plant pathogens now-a-days. Furthermore, AgNPs mediated controlling plant pathogens are environment-benign in comparison to chemically synthesized fungicides^28–30^. Thus, the AgNPs-mediated approach of controlling the pathogenic agricultural-microbes is both cost-effective and eco-friendly^31–33^. The researchers have suggested the various methods viz., chemical, physical, and biological methods for synthesis of nanoparticle but among these methods the phytomediated or green synthesis method is the most convenient, economical, and ecofriendly approach of nanoparticle synthesis^34,35^.

Moreover, when these AgNPs are interacted with PGPR bacteria, they may enhance the growth of microorganisms or inhibits them depending upon the concentration of nanomaterials added. Their unique size-dependent properties make these materials superior and indispensable in many areas of sustainable agriculture. Timmusk et al.^36^ reported that PGPR and nanoparticles significantly enhanced the growth of wheat plant and also inhibited the growth of *Fusarium culmorum*. Several other workers from different part of world also reported the effect of interactive study between PGPR and nanoparticles on different crops^25,37,38^. In this study, we isolated the *Bacillus cereus* LPR2 from the spinach rhizosphere and identified on the basis of the conventional biochemical and molecular methods. The bacterial species isolated from rhizospheric soil was also evaluated for its plant growth promoting ability such as IAA, HCN production, phosphate solubilization, and ammonia production. The isolated *Bacillus* species and phytomediated synthesized silver nanoparticle from *Tagetes erecta* were evaluated for plant growth activity individually as well as in combination.

## Material and methods

### Isolation and identification of rhizospheric Bacteria

Healthy and young spinach plants were gently uprooted from the fields of Chunni Kalan (Punjab) in the month of January–February and were transported to the laboratory in sterile zip-lock polythene bags. Each sample was stored in a refrigerator at 4 ^ο^C till further processing. The rhizospheric soil of the spinach plant was removed and air dried. 1 g of soil was used for serial dilutions. 0.1 ml of suspension from dilutions (10^–4^) was carefully spreaded on Nutrient Agar (HiMedia) plates, incubated at 28 ^ο^C for 24–36 h and observed for the appearance of distinct individual colonies. The pure cultures were stored at 4 °C for further use. All the isolates were primarily identified as *Bacillus* spp. on the basis of phenotypic (morphological and biochemical) characterization.

### Molecular characterization of efficient isolate

To identify the bacterial isolate LPR2, genomic DNA was isolated and 16S rRNA gene sequence was amplified. The gene sequence of the bacterial isolate was submitted to the NCBI to get accession number. Further, the gene sequence was used to compare the 16S rRNA gene sequence of bacteria using nBLAST tool in NCBI (www.ncbi.nlm.nih.gov/) database.

### Biosynthesis of silver nanoparticles from *Tagetes erecta*^39^

Step 1: Preparation of water extract of leaves and flowers of *T. erecta*.

**Figure 1:**
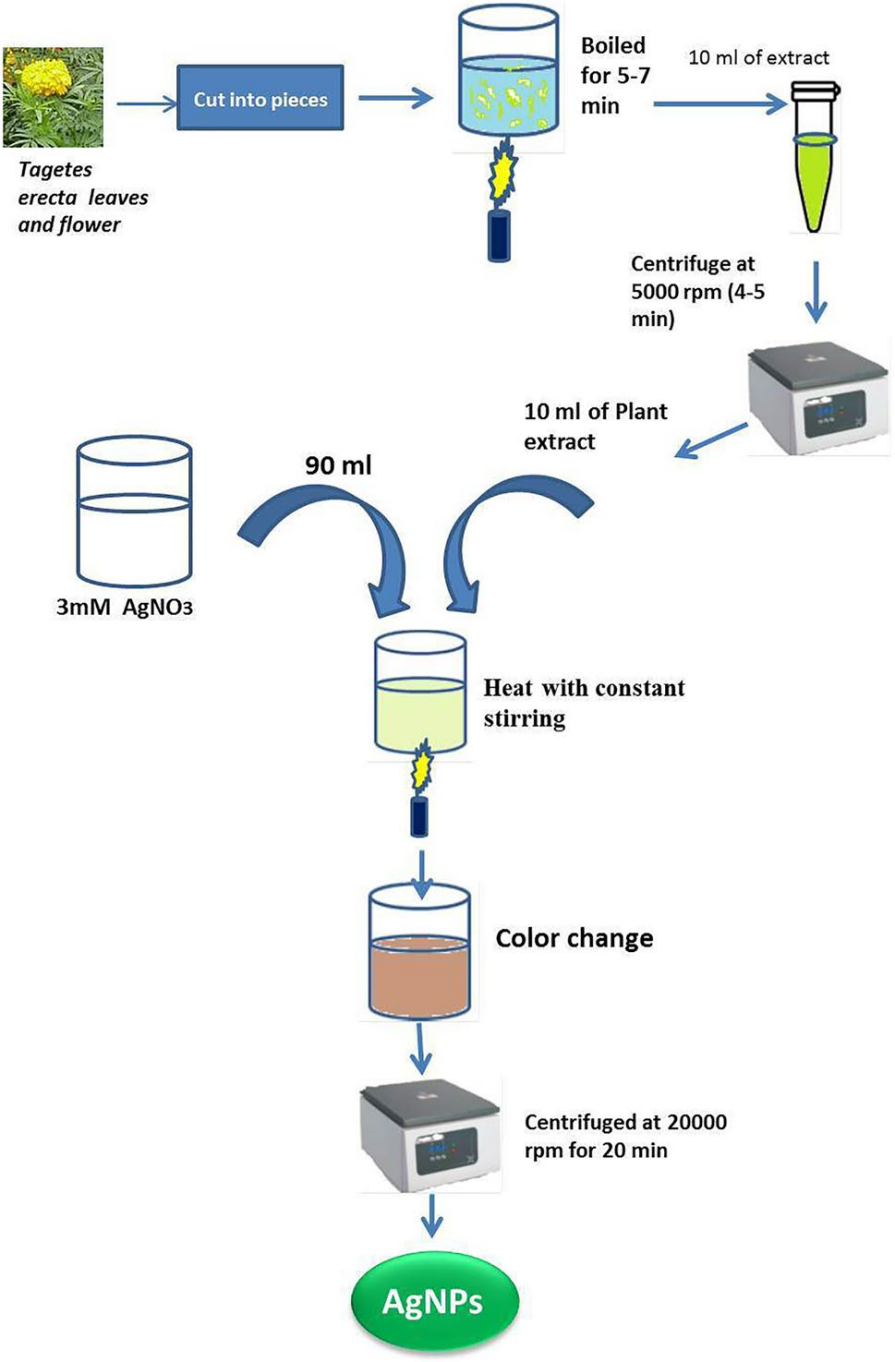
Preparation of silver nanoparticles (AgNPs) from leaves and flowers extract of *T.erecta*.

The fresh leaves and flower of wild and healthy marigold (*Tagetes erecta*) were collected and thoroughly washed in running tap water followed by washing in double distilled water (DDW)*.* 100 mg of finely cut leaves and flowers of marigold were added to 20 ml of DDW and boiled for 5–7 min with continuous stirring in a 100 ml conical flask. After boiling, 10 ml of the extract was taken in a 15 ml tube and centrifuge at 5000 rpm for 3–4 min to remove the plant debris (Fig. 1).

Step 2: Preparation of 30 mM silver nitrate (AgNO_3_) solution.

The stock solution of 30 mM silver nitrate (AgNO_3_) was prepared by dissolving 510 mg of silver nitrate in 100 ml of DDW. Finally, 3 mM solution of silver nitrate (AgNO_3_) was prepared from the stock solution.

Step3: Preparation of green silver nanoparticles.

The plant water extract (10 ml) was added to 90 ml 3 mM silver nitrate (AgNO_3_) solution with constant stirring for the bioreduction process. The colour changes to reddish brown which indicated the formation of silver nanoparticles. The complete reduction of Ag^+^ into AgNPs (Ag^0^) occurred within 25–30 min of reaction. Further, the solution was kept for an incubation period of 24 h at room temperature.

Step 4: Isolation of silver nanoparticles.

The solution containing silver nanoparticles was centrifuged at 20,000 rpm for 20 min. The supernatant was discarded and this step was repeated thrice and at last silver nanoparticles were suspended in 5 ml double distilled water.

### Characterization of nanoparticles

#### Characterization of silver nanoparticles using UV–Vis spectroscopy

The UV–Vis spectrometry analysis was done to characterize the synthesized AgNPs. The reduction of pure silver ions was observed by measuring the UV–Vis spectra of synthesized AgNPs in the wavelength range of 360 to 660 nm.

### Characterization of silver nanoparticles using High Resolution Transmission Electron Microscopy (HRTEM) and Energy Dispersive X-ray (EDX) spectroscopy

The HRTEM images were obtained with the help of an FEI TECNAI (G2 F20) system (200 keV) with a resolution of 0.2 Å and 6 × 10^6^ times magnification. The ingredient element of synthesized NPs was also analyzed using EDX spectroscopy on the same machine^40^.

### Preparation and seed treatment with Ag nanoparticles

For the preparation of 200 ppm of AgNP solution, 0.2 mg of stock solution was diluted in 100 ml of distilled water. The solution was further diluted to 25 ppm and applied as seeds soaking treatment for 3 h prior to sowing. The aqueous solution of AgNPs (50 ppm) was also sprayed on the plants 6 days after seeds sowing.

### Characterization of PGPR attributes

#### IAA production

The bacterial culture was inoculated in Nutrient Broth (HiMedia) containing tryptophan (0.1 g/l). The exponentially grown culture was centrifuged at 10,000 rpm at 4 °C for 15 min. The supernatant (2 ml) was mixed with two drops of *O*-phosphoric acid and 4 ml of Salkowski’s reagent (1 ml of 0.5 M FeCl_3_ in 50 ml of 35% HClO_4_). The development of pink colour confirmed the production of IAA^41^.

### Phosphate solubilization

The phosphate solubilization activity of isolates was detected by spotting the isolates on Pikovskaya’s Agar (HiMedia) plates^42^. The plates were incubated at 30 °C for 2 days and then observed for the appearance of a clear halo zone around the colonies due to solubilization of inorganic phosphate by organic acid produced by bacteria.

### HCN production

The HCN production was determined by the modified method of Bakker and Schippers^43^. The young culture of the isolate was streaked and inoculated on an agar plate containing 0.44% glycine and covered with filter paper already soaked in 0.5% of picric acid in 1% Na_2_CO_3._ The plate was sealed with parafilm and incubated at 30 °C for 48–72 h for the observation of color change from yellow to light, moderate and strong brown in the filter paper due to the production of HCN by bacteria.

### Ammonia production

Fresh peptone water was used to test the production of ammonia by the isolate. 48 h old culture of each isolates was inoculated in 10 ml of peptone water and incubated at 30 °C for 72 h. 0.5 ml of Nessler’s reagent was added in each tube and after few minutes yellow to brown precipitate appeared which indicates the production of ammonia^44^.

### Antagonistic activities

The dual culture technique was used to measure the activity of the isolated bacterial strain against the fungal pathogen *Macrophomina phaseolina*, a causal agent of charcoal rot in maize, procured from the culture collection center, Department of Microbiology, Dolphin College, Chandigarh*.* Agar block (5 mm in diameter) from the margin of 5 days old culture of the fungal pathogen was placed in the centre of the assay plate. One loopful (24 h old) culture of bacterial isolates was spotted 2 cm apart from the pathogenic fungus. Petri plate inoculated by the pathogen in the center but without bacterial isolates was used as control (positive). All the plates were incubated at 28 °C for 3–7 days. The zone of inhibition (%) was recorded by using the following formula:

$${\text{Inhibition}}\,{\text{zone }}\left( \% \right) = \left[ {{\text{C}} - {\text{T}}/{\text{C}}} \right] \times {1}00$$

where C is radial growth of fungus in the control plate; T is the radial growth of fungus in the test plate.

### Seed bacterization

The seeds of maize were collected from the local market of Chunni Kalan, Fatehagarh Sahib, Punjab, (India). Healthy seeds of similar shape and size were selected for the study and 4 seeds per pot were taken into sterile Petri plates. The bacterization of seeds in Petri plate was done aseptically under a Laminar Air flow cabinet. The selected seeds were washed with sterilized distilled water followed by 2–3% NaOCl (sodium hypochlorite) for 2–3 min. Further, seeds were washed with 70% ethanol for 2–3 min and then rewashed with double distilled water for 2–3 times to remove the traces of NaOCl and ethanol and dried under laminar hood in Petri plates. The bacterial strain *B. cereus* LPR2 was grown in Nutrient Broth (HiMedia) for 48 h at 28 °C in a shaker incubator. The culture of bacterial strain was mixed separately with 1% Carboxymethyl cellulose (CMC) solutions to form slurry. This slurry was used to coat the surface of sterile seeds (maize) by putting the seeds in a mixer of CMC slurry and bacterial broth for 1 h individually and in combination with AgNPs. The seeds were also treated with AgNPs by dipping in 25 ppm solution of AgNPs for 3 h before sowing the seeds and sprayed by 50 ppm solution of AgNPs after 6 days of seed germination.

### Pot assay

The sterilized garden soil was used for pot assay. The garden soil was filled in tin box and formalin was poured inside it. This was left undisturbed for 7 days at room temperature; gradually soil was transferred to the pot. The bacterized seeds were sown in 4 pots (4 seeds per pots). The moisture of soil was maintained using the regular use of sterile water. The plants were uprooted after 10 days of sowing for the measurement of plant growth parameters like plant length and fresh and dry weight. Treatments were as follow:—T1: seeds bacterized with LPR2, T2: seeds coated with AgNPs, T3: seeds bacterized with LPR2 + AgNPs, T4: seeds of maize coated with 1% CMC slurry only as control.

### Statistical analysis

The data were analyzed by using the analysis of variance applying ANOVA software.

## Results

### Isolation and identification of rhizospheric bacteria

The rhizobacteria were isolated from spinach by serial dilution method using Nutrient Agar Medium, and characterized on the basis of their phenotypic characters. Out of several isolates, only five (LPR1 to LPR5) isolates were selected (Table S1) for the further study. Based on good in vitro plant growth promoting activities the isolate LPR2 was selected for pot assay. Molecular characterization of the isolate LPR2 revealed that 16S rRNA gene sequences of the isolate were similar to *Bacillus* spp. available in NCBI data base and was identified as *B. cereus* LPR2 (Accession number MH997647.1).

### Structural and morphological analysis of AgNPs

**Figure 2:**
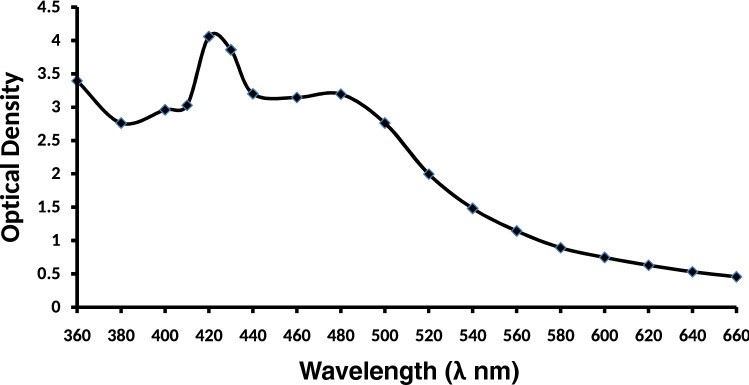
UV–Vis absorption spectrum of silver nanoparticles synthesized from *Tagetes* erecta.

The UV–Vis spectroscopy was used to characterize the AgNPs obtained from *T. erecta*. The Plasmon absorbance was found to be near 420 nm which is an attribute of phytosynthesized AgNPs (Fig. 2).

### HRTEM and EDX analysis of AgNPs

**Figure 3:**
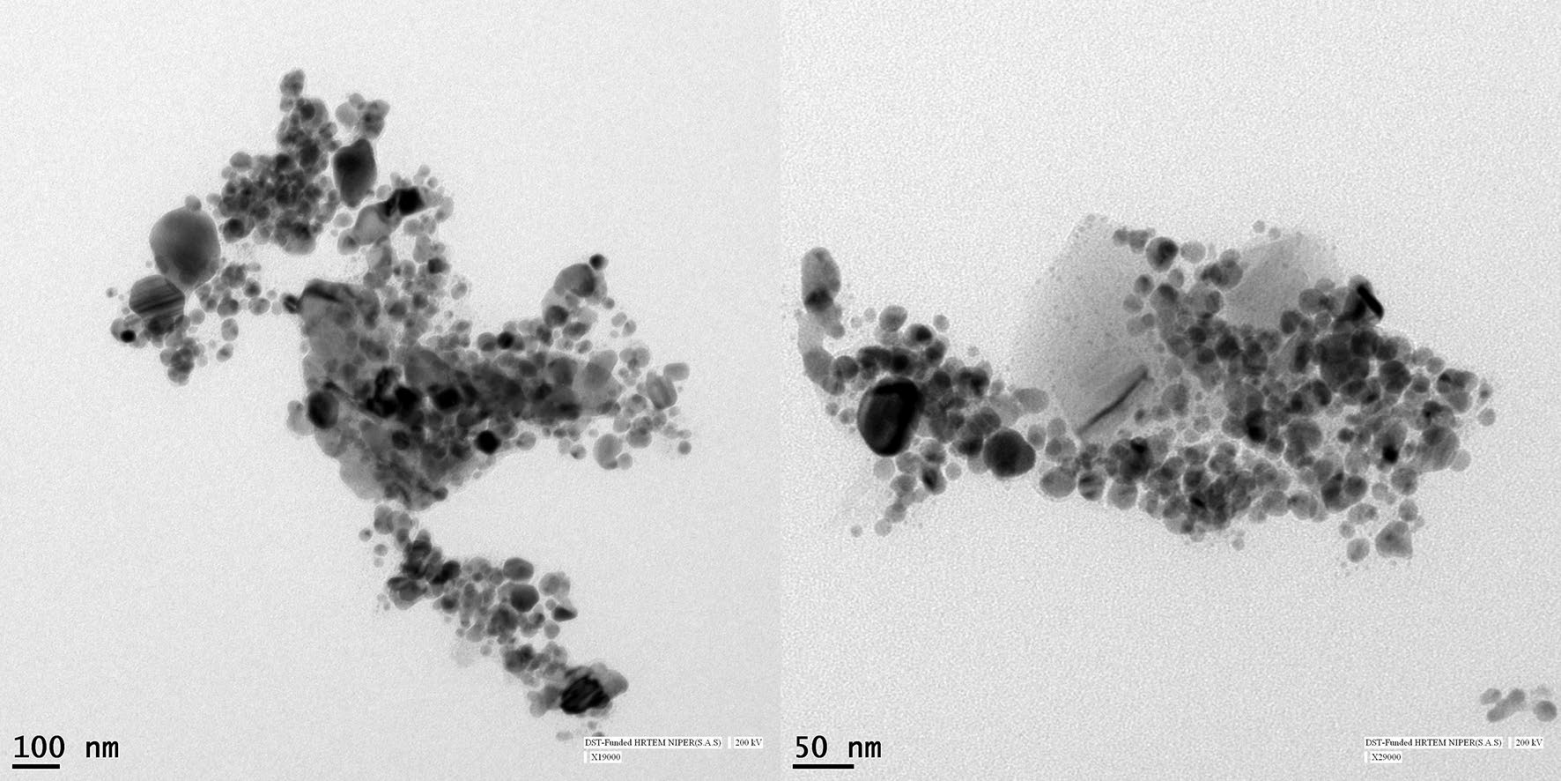
HRTEM photograph showing silver nanoparticles (AgNPs). Maximum nanoparticles have size less than 60 nm.

**Figure 4:**
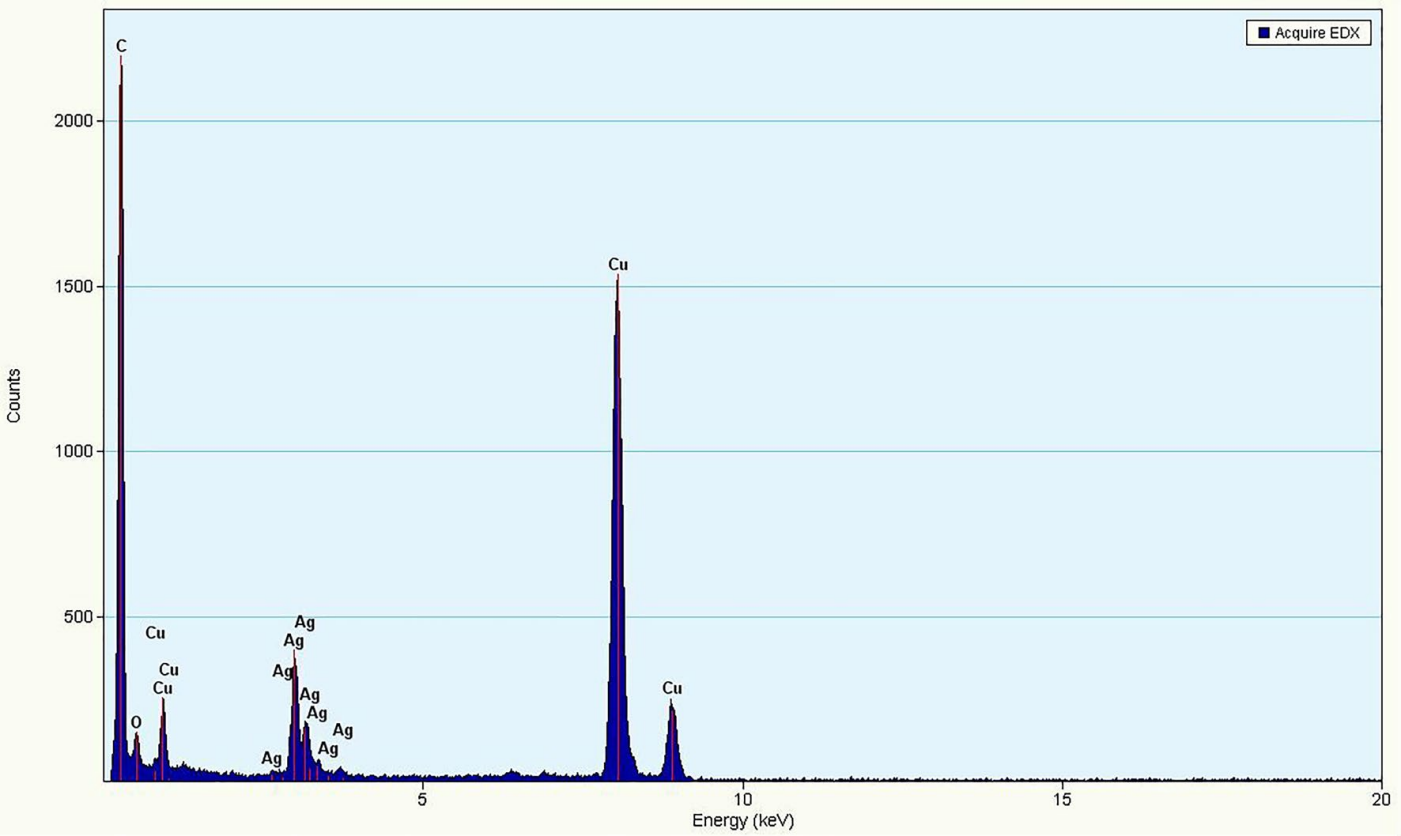
EDX spectra of phytosynthesized AgNPs.

The High Resolution Transmission Electron Microscopy revealed that the AgNPs are mostly of oval shaped and have a wide range of size particles ranging from 20 to 60 nm (Fig. 3), whereas Energy Dispersive X-ray (EDX) analysis confirmed the presence of only silver metal as AgNPs in the given reaction (Fig. 4).

### Characterization of isolates for PGP attributes

All the selected five isolates were tested for IAA, HCN, ammonia production and phosphate solubilization activities. Several isolates showed good activities but LPR2 very efficiently produced IAA, HCN and ammonia, and was able to solubilize phosphate forming the large halo zone in comparison to the other isolates (Table 1, Fig. S1).

**Table 1:** Plant growth promoting attributes and antagonistic activities of *Bacillus cereus* LPR2 and other isolates.

PGPR attributes	LPR1	LPR2	LPR3	LPR4	LPR5
IAA production	+	++	++	+	+
Phosphate solubilisation	++	+++	+	++	+
HCN Production	+	++	++	++	+
Ammonia Production	+	++	+	+	++
Antagonistic activities against *Macrophomina phaseolina*	−	+++	++	++	−

*Abbreviation* (+) positive; +, ++,+++ production and solubilization in increasing order; Antagonistic activities -, do not inhibit pathogen, +, ++, +++ inhibit the growth of pathogen (in increasing order).

### Antagonistic activities

**Figure 5:**
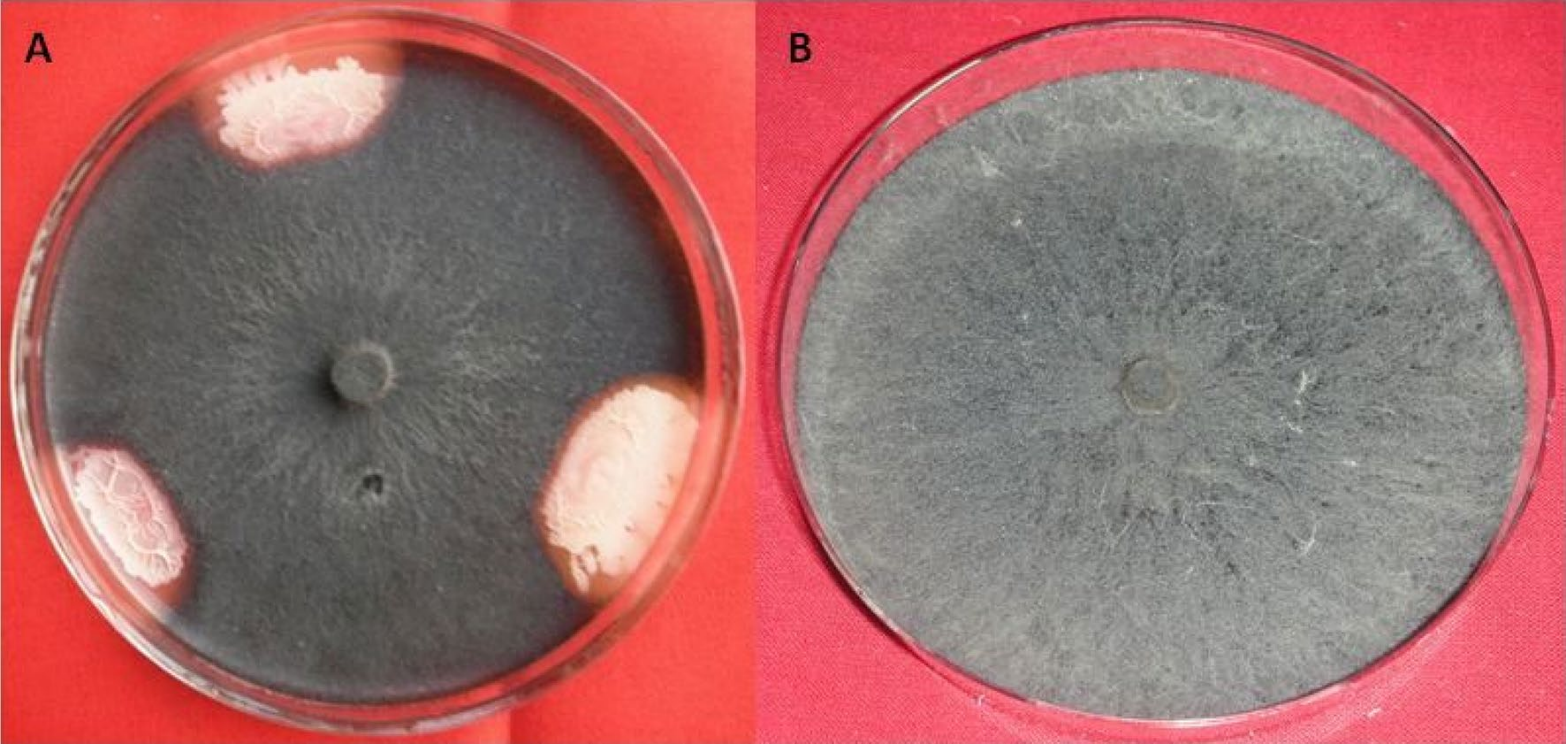
Antagonistic activities of *Bacillus cereus* LPR2 against *Macrophomina phaseolina,* Test plate (**A**) and Positive Control plate (**B**).

All the isolates were also screened for their antagonistic activities. Fungal growth inhibition based on average zone of inhibition, by rhizobacterial isolates, was measured in millimeter (mm) which was the highest with LPR2 (16.2 mm) followed by LPR3 (14.6 mm) and LPR5 (11.7 mm). The average radial growth (in mm) was also recorded to calculate the inhibition and found that isolate LPR2 was able to inhibit 72% the growth of *M. phaseolina* after 7 days of incubation (Table 1, Fig. 5a, b).

### Pot trial studies

Maize seeds of uniform shape and size were coated with the *B. cereus* LPR2, AgNPs, and LPR2 + AgNPs. Seeds coated with the LPR2 microbial inoculant, AgNPs, and combination of LPR2 + AgNPs showed the induced vegetative parameters after 10 days of inoculation. Maximum seed germination, and shoot and root length were observed with *B. cereus* LPR2 followed by the treatment with AgNPs. But the combination of LPR2 + AgNPs did not show the significant results in comparison with seeds treated with LPR2 and AgNPs. Similar trends of enhancement were obtained with shoot fresh and dry weight and root fresh and dry weight of plant treated with LPR2 followed by the AgNPs. All the parameters increased and were significant at CD 1% and CD 5% as compared to control (Table 2). The maize seeds bacterized with LPR2 and treated with AgNPs showed a significant increase in seed germination (87.5%) followed by the combination of the LPR2 + AgNPs (75%). In the control treatment, seed germination was 50%. The significant enhancement of the germination was observed after treatment of the maize seeds; all germination parameters were enhanced at higher AgNPs dosages. It is probable that nanoparticles penetrated the seed coat and exerted a beneficial effect on the process of seed germination. Based on the studies of nanoparticle effects on seed germination mechanisms, possibly nanoparticles mediates to increase water absorption by the seeds.

**Table 2:** Effect of *Bacillus cereus* LPR2 and silver nanoparticles on seed germination and growth parameters of *Zea mays*.

Isolates	Seed Germination (%)	Root length (cm)	Shoot length (cm)	Root weight (g)		Shoot weight (g)	
				Fresh wt	Dry wt	Fresh wt	Dry wt
LPR2	87.5%	12.567**	12.500**	1.1967**	0.257**	0.647**	0.140**
AgNPs	87.5%	10.767**	10.066**	1.067**	0.167*	0.520**	0.090**
LPR2 + AgNPs	75%	10.367**	9.133^ ns^	0.783^ ns^	o.113^ ns^	0.460**	0.073^ ns^
Control	50%	8.901	8.500	0.623	0.085	0.413	0.050
SEM		0.195	0.191	0.049	0.022	0.009	0.008
CD 1%		1.018	0.994	0.255	0.115	0.049	0.040
CD 5%		0.674	0.658	0.169	0.076	0.032	0.027

SEM= standard error mean; CD= Critical Difference, Values are mean of 3 randomly selected plants from each set, ** significant 1%, * significant at 5% as compared to control, ns= non-significant as compared to control, Control (Non-bacterized seeds).

## Discussion

We all are aware of the fact that the world population is expanding exponentially day by day, which will need more good quality of food for their survival but the food production is not increasing accordingly due to the several constraints. To combat with this problem, several workers across the globe are trying to develop the advanced technologies that can enhance the production of crops^45^, and nanotechnology is one of them. Although this approach is eco-friendly but some reports reveal that nanoparticles, in general, have both negative and positive effects on the growth of plants^46^. The nanotechnology has shown immense potential in biomedicals, physics, electronics and chemical sciences. However, no much work has been done in the field of agricultural sciences. There are several reports which advocated the benefits of synthesized nanoparticles in term of plant growth promotion and disease suppression. A study was carried out by Chavan and Nadanathangam^25^ in which they applied the zinc and silver nanoparticles directly into the soil and monitored the effect of these nanoparticles on the growth and microbiome of soil. The result showed a feeble effect on the growth of bacterial species. In contrast, foliar spray of silver nanoparticle on *Trigonella foenum-graecum* increased most of the growth parameters and other biochemical profile^47^.

In this study, we have isolated several rhizobacteria from spinach and on the basis of in vitro plant growth promoting activities of isolates, LPR2 was found the most potent PGPR having the ability to solubilize tricalcium phosphate, produce IAA, HCN, and ammonia. Besides these growth promoting activities, the LPR2 was also able to show inhibitory effect against the growth of soil borne phytopathogen *M. phaseolina.* On the basis of 16S rRNA gene sequencing, the isolate was identified as *Bacillus cereus* LPR2. Similarly, several workers across the globe isolated the numerous potential plant health promoting and antagonistic microbes like *Azotobacter* spp., *Bacillus* spp., *Klebsiella* spp., *Pseudomonas* spp., *Streptomyces* spp., etc. from different types of plants^13,20,22,48,49^.

In this study, AgNPs obtained from *T. erecta* were characterized by using UV–Vis spectroscopy and the plasmon absorbance was recorded near 420 nm. Other techniques such as HRTEM used for the analysis of shape and size of nanoparticles revealed that the AgNPs are mostly of oval shaped and particles size varied from 20 to 60 nm, whereas Energy Dispersive X- ray analysis confirmed the presence of only silver metal as AgNPs in our sample^50–52^. Due to the small size of nanoparticles they can easily transfer to a cell or tissue according to need and help in the delivery of required molecules to plants. The permeability of cell wall and some carrier protein present in the cell wall of plant cells help in the entry of nanoparticles inside the plant cell. Although nanoparticles have several advantages in relation to size and easy to deliver in cell or tissue yet various workers have reported different effects *i.e.* positive, negative, and neutral effects of nanoparticles on plant growth parameters and seed germination^25,27,53^.

The maize seeds coated with the *B. cereus* LPR2, AgNPs, and LPR2 + AgNPs were used for pot trial in this experiment. Seeds coated with the LPR2 microbial inoculant, AgNPs and combination of LPR2 + AgNPs showed the induced vegetative parameters after 10 days of inoculation. Maximum seed germination, and shoot and root length were observed with *B. cereus* LPR2 followed by the treatment with AgNPs. But the combination of LPR2 + AgNPs did not show the significant results in comparison with seeds treated with LPR2 and AgNPs separately. It is probable that nanoparticles penetrate the seed coat and exert a beneficial effect on the process of seed germination. On the basis of nanoparticle effects on the mechanism of seed germination, it is possible that nanoparticles help to increase water absorption by seeds. As per the evidence from the findings of other workers, the combination of LPR2 and nanoparticles (AgNPs) in our study did not enhance the growth of maize in comparison to LPR2 and nanoparticles (AgNPs) individually meaning that the nanoparticles may be beneficial/ detrimental/ neutral for plant and sometimes nanoparticles may be detrimental for PGPR also. In a study, treating castor seeds with AgNPs did not show any effect on seed germination^54^. Nawaz and Bano^53^ reported that AgNPs were not effective in combination with PGPR because it inhibited the efficiency of PGPR. This type of findings have also been reported by several researchers in different plants, such as soybean, chickpea, maize, cucumber and mustard seeds^55^^–^^57^. Gruyer et al.^58^ also observed that AgNPs can induce or inhibit the plant parameters in different crops. In the case of barley, the root length was increased whereas in lettuce it was inhibited^59^.

Thus, it might be concluded that the bacterial strain *B. cereus* LPR2 with its multifunctional properties will attract more attention in the field of bio-fertilization and biological control. The present investigation revealed the ability of *B. cereus* LPR2, having good plant growth promoting attributes such as phosphate solubilization, IAA production, HCN production, and ammonia production and biocontrol. AgNPs also enhanced seed germination, root length in comparison to control but without the effect on plant growth in combination with *B. cereus* LPR2. So, the isolated bacterial strain *B. cereus* LPR2 and AgNPs separately could be used as bioinoculant individually for maize and other crops. The economy of India is agricultural based and its population is the second highest after China. The demand of food grains, vegetable, etc. is increasing day by day and the reason of decreased productivity may be due to the microbial infestation and insect/pests. The Indian government runs different projects and programmes to train the farmers to divert their routine agriculture, which is totally based on chemical fertilizer and pesticide, to biofertilizer, biopesticide, and antagonistic microorganism. Thus our study is one step towards the green approach to combat with microbial infestation and to increase crop productivity by using nanoparticles.

## Supplementary information

Supplementary information.

## Data Availability

The authors declare that all the data supporting the findings of this study are available within the article and from the corresponding authors on reasonable request.
