# Supplementary material for: Effect of silver nanoparticles and Bacillus cereus LPR2 on the growth of Zea mays
Source: Sci Rep. 2020 Nov 23;10:20409. doi: 10.1038/s41598-020-77460-w (PMC7683560; doi:10.1038/s41598-020-77460-w)
Supplement: Supplementary file 1 — Supplementary information. [file 41598_2020_77460_MOESM1_ESM.docx]

**Supplementary information**

**Effect of Silver nanoparticles and *Bacillus cereus* LPR2 on the growth of *Zea mays***

**Pankaj Kumar^a,b*^, Vikas Pahal^a^, Arti Gupta^c^, Ruchi Vadhan^a^, Harish Chandra^d*^, Ramesh Chandra Dubey^d^**

*^a^Department of Microbiology, Dolphin (PG) College of Science and Agriculture, Fatehgarh Sahib, Chandigarh, 140307, Punjab, India*

*^b^Department of Microbiology, Dolphin (PG) Institute of Biomedical and Natural Sciences, Dehradun, 248007, Uttarakhand, India*

*^c^Department of Zoology, Sri Avadh Raj Singh Smarak Degree College, Gonda, Uttar Pradesh, India*

*^d^Department of Botany and Microbiology, Gurukula Kangri Vishwavidyalaya, Haridwar 249404, Uttarakhand, India*

*Corresponding author e-mail:

Dr Pankaj Kumar ([*guptapankaj23@gmail.com*](mailto:guptapankaj23@gmail.com)),

Dr. Harish Chandra ([*hreesh5@gmail.com*](mailto:hreesh5@gmail.com))

**Running head:** Silver nanoparticle and PGPR

**Table S1: Morphological and biochemical characterization of bacterial isolates (LPR1 to LPR5) of spinach rhizosphere**

| **Parameters** | **ISOLATES** | | | | | |
| --- | --- | --- | --- | --- | --- | --- |
|  | **LPR 1** | **LPR 2** | **LPR 3** | **LPR 4** | **LPR 5** | **MTCC 8528** |
| Gram Staining | **+** | **+** | **+** | **+** | **+** | **+** |
| Shape | **rod** | **rod** | **rod** | **rod** | **rod** | **rod** |
| Motility | **+** | **+** | **+** | **+** | **+** | **+** |
| Catalase Test | **+** | **+** | **+** | **-** | **+** | **+** |
| Indole Test | **-** | **-** | **-** | **-** | **+** | **-** |
| Starch hydrolysis | **+** | **+** | **+** | **-** | **-** | **+** |
| Citrate utilization | **+** | **+** | **+** | **+** | **-** | **+** |
| Methyl red | **-** | **-** | **-** | **+** | **-** | **-** |
| Voges Proskaur’s | **+** | **+** | **+** | **-** | **+** | **+** |
| Oxidase | **-** | **+** | **-** | **+** | **-** | **-** |
| Phenylalanine deamination | **-** | **-** | **-** | **-** | **-** | **-** |
| Nitrate reduction | **+** | **+** | **+** | **-** | **+** | **+** |
| Lysine decarboxylase | **+** | **+** | **+** | **-** | **-** | **+** |
| Urease | **-** | **-** | **-** | **-** | **-** | **-** |

**Abbreviations:**(+) positive and (-) negative; MTCC 8528*, Bacillus subtilis* (standard known culture procured from MTCC (Microbial Type Culture Collection, Chandigarh, India) used to compare the results with our isolates for identification on the basis of phenotypic parameters)

**
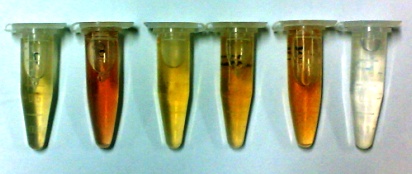

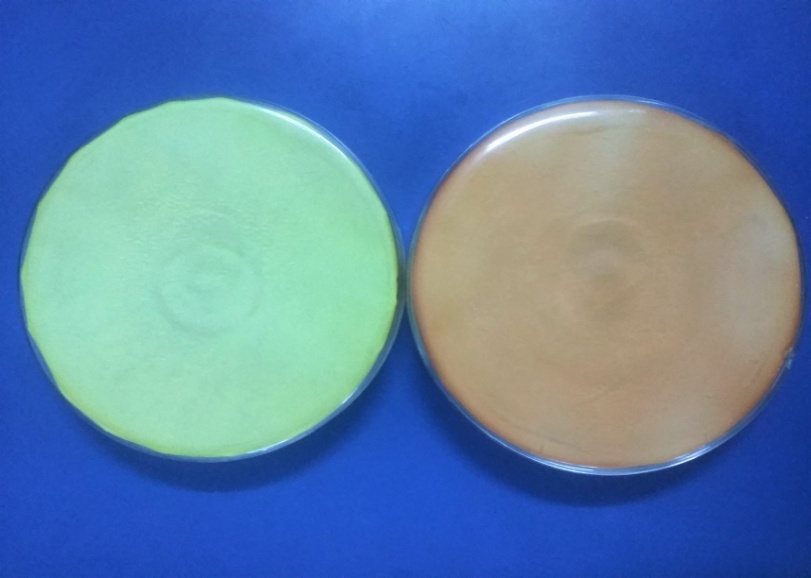

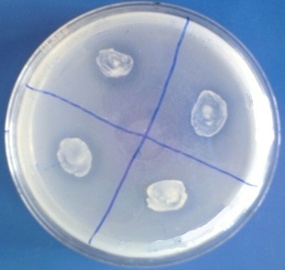
**

**Figure S1: Plant Growth Promoting attributes of *Bacillus sp.* isolated from spinach rhizosphere: Phosphate solubilization (A), Indole acetic acid production (B), HCN production (C), Ammonia production (D).**

**B**

**Control (HCN)**

**D**

**C**

**A**

**A**
